# Repression and Recuperation of Brood Production in *Bombus terrestris* Bumble Bees Exposed to a Pulse of the Neonicotinoid Pesticide Imidacloprid

**DOI:** 10.1371/journal.pone.0079872

**Published:** 2013-11-04

**Authors:** Ian Laycock, James E. Cresswell

**Affiliations:** 1 College of Life & Environmental Sciences, Biosciences, University of Exeter, Exeter, United Kingdom; 2 Centre for Pollination Studies, University of Calcutta, Kolkata, India; French National Institute for Agricultural Research (INRA), France

## Abstract

Currently, there is concern about declining bee populations and some blame the residues of neonicotinoid pesticides in the nectar and pollen of treated crops. Bumble bees are important wild pollinators that are widely exposed to dietary neonicotinoids by foraging in agricultural environments. In the laboratory, we tested the effect of a pulsed exposure (14 days ‘on dose’ followed by 14 days ‘off dose’) to a common neonicotinoid, imidacloprid, on the amount of brood (number of eggs and larvae) produced by *Bombus terrestris* L. bumble bees in small, standardised experimental colonies (a queen and four adult workers). During the initial ‘on dose’ period we observed a dose-dependent repression of brood production in colonies, with productivity decreasing as dosage increased up to 98 µg kg^−1^ dietary imidacloprid. During the following ‘off dose’ period, colonies showed a dose-dependent recuperation such that total brood production during the 28-day pulsed exposure was not correlated with imidacloprid up to 98 µg kg^−1^. Our findings raise further concern about the threat to wild bumble bees from neonicotinoids, but they also indicate some resilience to a pulsed exposure, such as that arising from the transient bloom of a treated mass-flowering crop.

## Introduction

Currently, there is concern about declines in bee populations [1], [2] and some implicate neonicotinoid pesticides as culprits [3], [4]. Neonicotinoids disrupt the insect nervous system [5] and their dietary intake can reduce the expected performance of bees [6], [7]. For example, neonicotinoids may increase worker losses while reducing reproductive output and foraging performance in bumble bees, *Bombus* spp. [8], [9], and induce homing failure and suppress colony growth in honey bees, *Apis mellifera* L. [10] (and see [11], [12] for further discussion). Whether neonicotinoids are a principal cause of bee declines is unclear [13], [14], but in regions where they are not banned [4] bees are certainly exposed to them on a massive spatial scale by foraging from treated agricultural crops. For example, oilseed rape (or canola), *Brassica napus* L., is the principal mass-flowering crop in many areas of North America (>8 million hectares [15], [16]) and Northern Europe (e.g. ∼0.7 million hectares in the UK [17]) and many of its fields are protected from pests by neonicotinoids [18], [19]. Neonicotinoids are systemic pesticides, so they are distributed throughout the plant following application [18] and bees are exposed to dietary residues by consuming nectar and pollen [20]. For oilseed rape in the USA, residues of a widely used neonicotinoid, imidacloprid, have been detected in nectar at 0.8 parts per billion (ppb) and in pollen at 7.6 ppb [21]. Other bee-attractive crops such as sunflower and alfalfa are often protected with neonicotinoids [18], [21], and so the exposure of bees to these pesticides is widespread. To understand whether a widespread exposure to neonicotinoids is capable of causing bee populations to decline, we must understand their demographic toxicity, which occurs when a toxic agent detrimentally affects the birth and death rates of the exposed species [22].

The lethality of imidacloprid to bees appears to be dependent on the time of exposure [23], [24]. However, in some laboratory trials the trace levels of imidacloprid typically found in nectar and pollen (≤10 ppb [21], but see [25]) have negligible effects on mortality in honey bees [7] and bumble bees [26], [27], but they can substantively affect birth rates in bumble bees [28]. Specifically, dietary imidacloprid at levels as low as one ppb may reduce the number of eggs and larvae produced by adult bumble bee workers by one third [28], but the demographic implications of this are unclear because queens are principally responsible for a colony's reproductive output [29]. Because the number of new queens and males that a bumble bee colony produces depends on its size [30], [31], the number of workers produced by a queen during a colony's development can determine colony fitness. We therefore examined the effects of dietary imidacloprid on brood production (specifically, the numbers of eggs and larvae destined to become workers) by queen bumble bees at dosages that spanned the environmentally realistic range.

We investigated the effects of a 14-day exposure to dietary imidacloprid on the performance of small, standardised experimental colonies of the buff-tailed bumble bee, *Bombus terrestris* L., in the laboratory. We found a dose-dependent decrease in brood production up to 98 ppb imidacloprid (see Results) and so we extended our experiment to create a pulsed exposure, feeding bees for an additional 14 days on an imidacloprid-free diet, because a scenario such as this may be relevant to wild bumble bee colonies. For example, a pulsed exposure may be caused by the synchronized bloom of imidacloprid-treated oilseed rape fields that normally flower for approximately four weeks in April or May [32] (where the crop is winter-sown) and the exposure subsides when the bees subsequently switch to foraging on pesticide-free wildflowers [33]. Recuperation from some imidacloprid-induced effects has been reported following an exposure in honey bees [34], coccinellids [35], aphids [36], whitefly [37], and the aquatic larvae of midge [38], but our study is the first to explore the potential for such a recovery in bumble bees.

## Materials and Methods

### Ethics statement

The protocol reported here conforms to the regulatory requirements for animal experimentation in the UK and was approved by the Biosciences Ethics Committee at the University of Exeter.

### Bees, experimental colonies and imidacloprid diets

We obtained colonies of *B. terrestris* (subspecies *audax*) at an early stage of development (Biobest, Westerlo, Belgium). In order to create small, standardised experimental colonies for testing, we removed each queen and randomly chose four of her adult workers from their pre-experimental source colony and placed them together in a softwood box (120×120×45 mm) fitted with two 2 mL microcentrifuge tubes (Simport, Beloeil, Canada) that were punctured so as to function as syrup (artificial nectar) feeders [28]. Experimental colony size (a queen and four adult workers) was chosen to simulate early-stage bumble bee colonies, consistent with those used in similar studies [8]. We maintained these experimental colonies for 28 days in a semi-controlled environment (23–27°C, 21–47% relative humidity).

We obtained imidacloprid as a solution in acetonitrile (Dr. Ehrenstorfer GmbH, Ausberg, Germany). Acetonitrile was removed by evaporation and the imidacloprid was dissolved in purified water before being mixed into feeder syrup (Attracker: 1.27 kg L^−1^ fructose/glucose/saccharose solution; Koppert B.V., Berkel en Rodenrijs, Netherlands) to produce our most concentrated dosage of 125 µg imidacloprid L^−1^ (or 98.43 µg kg^−1^ = ppb). By serial dilution from 125 µg L^−1^ (dilution factor = 0.4) we produced the following nine experimental dosages: 125.00, 50.00, 20.00, 8.00, 3.20, 1.28, 0.51, 0.20, and 0.08 µg imidacloprid L^−1^ ( = 98.43, 39.37, 15.75, 6.30, 2.52, 1.01, 0.40, 0.16, and 0.06 µg imidacloprid kg^−1^). A fresh dilution series containing all nine concentrations was produced at the beginning of each pulsed exposure trial (see below) and kept inside a dark fridge at 5°C. Dosed syrup from the second pulsed exposure trial was used in the continuous exposure experiment (below).

### Exposure to dietary imidacloprid

To create a pulsed exposure, the 28-day experimental period was split into two successive periods of 14 days. During the ‘on dose’ period (days 1–14), 60 experimental colonies were provided *ad libitum* with either undosed control syrup (6 control colonies) or dosed syrup (6 colonies per dosage treatment, listed above). Fresh syrup at the appropriate dosage was provided to colonies daily. For the ‘off dose’ period (days 15–28), the bees were transferred to new softwood boxes and fed *ad libitum* with only undosed control syrup. At the beginning of each 14-day period, each experimental colony was provided with a fresh ball of undosed pollen (Biobest, Westerlo, Belgium) to which bees had *ad libitum* access. Pollen balls (mean mass = 6.1 g, SE = 0.02) were prepared from ground pollen pellets mixed with water to form dough and were weighed before and after placement in colonies to quantify pollen consumption. We corrected for evaporation of water from syrup and pollen based on the mass change of several feeders and pollen balls kept in empty colony boxes under experimental conditions. Experimental colonies were kept in darkness except when monitored daily for the appearance of wax covered egg cells (indicating that oviposition had occurred), syrup consumption and individual mortality. To minimise disturbance to bees, we assayed brood production by collecting all laid eggs and larvae from experimental colony boxes only at the end of each 14-day period, (i.e. on days 14 and 28). The experiment was conducted in two replicate trials, one between October–November 2011 and the other between January–February 2012. Each trial comprised 30 experimental colonies and treatment groups were equally represented in both (3 colonies per treatment).

To establish that the observed recuperation from imidacloprid-induced effects under pulsed exposure (see Results) was caused by the removal of dietary imidacloprid rather than from acclimation to exposure over elapsed time, we conducted a separate continuous exposure experiment. Using the same husbandry techniques described above, we randomly assigned 12 experimental colonies to either 28 days feeding on control syrup (7 colonies) or 28 days feeding on syrup dosed at 98.43 µg imidacloprid kg^−1^ (5 colonies) and we used the same interruption to collect brood on days 14 and 28. This continuous exposure trial was conducted between March–April 2012. This protocol is an adequate test because the highest level of recuperation was observed at 98.43 µg kg^−1^ in the previous experiments (see Results).

To verify the concentration of imidacloprid in our doses, we first dissolved the dosed syrup in liquid chromatography-mass spectrometry (LCMS)-grade water (Fisher Scientific UK Ltd, Loughborough, UK) spiked with a reference standard of imidacloprid-d_4_ (Dr. Ehrenstorfer GmbH, Augsburg, Germany) at 100 µg L^−1^ (ratio of syrup to water = 5∶7). We used solid phase extraction (SPE) to extract imidacloprid and imidacloprid-d_4_ from the syrup as follows. Diluted dosed syrup samples were processed through 1 mL Discovery® DSC-18 SPE tubes (Sigma-Aldrich, Gillingham, UK) under positive pressure. We first conditioned the SPE tube with 1 mL pure LCMS-grade methanol (Fisher Scientific UK Ltd, Loughborough, UK) followed by 1 mL pure LCMS-grade water. A 1 mL sample was passed through the tube, before the tube was washed with 1 mL pure LCMS-grade water and the imidacloprid was eluted from the column with three separate, but equivalent, aliquots of pure LCMS-grade methanol totalling 450 µL. We removed the methanol by evaporation and the remaining imidacloprid was dissolved in 500 µL of pure LCMS-grade water. Imidacloprid samples were analysed in an Agilent 1200 series liquid chromatograph interfaced via an electrospray ionisation source to an Agilent 6410 triple quadrupole mass spectrometer (Agilent Technologies, Santa Clara, CA, USA) using methods described in Laycock et al. [28]. The instrument response was linear over the range 0.06–125 µg L^−1^ for imidacloprid and imidacloprid-d_4_ and we found that dosages in all trials contained appropriate levels of imidacloprid (pulsed exposure trial 1, *measured imidacloprid* = 0.989 × *nominal dosage* + 0.204, *R^2^*>0.99; pulsed exposure trial 2 and continuous exposure trial, *measured imidacloprid*  = 1.035 × *nominal dosage* − 0.205, *R^2^*>0.99).

### Statistical analyses

In our analyses, ‘brood’ represents the total number of eggs and larvae produced in an experimental colony in a given period. We tested whether the ‘brood’ dose-response relationships differed between our two pulsed exposure trials by analysis of covariance (ANCOVA), with ‘dosage’ (dosage of imidacloprid in µg kg^−1^) log-transformed to log(‘dosage’ + 1) as the covariate and ‘trial’ as the fixed factor, and detected no significant difference between the two trials and so the data were pooled for further analysis (ANCOVA: ‘on dose’ brood, dosage × trial, *F*_1, 56_ = 0.99, *P* = 0.32; ‘off dose’ brood, dosage × trial, *F*_1, 56_ = 0.03, *P* = 0.86; total brood, dosage × trial, *F*_1, 56_ = 0.34, *P* = 0.56). The size of the pre-experimental source colony (mean number of workers  = 16.4, SE = 1.1; mean number of brood  = 101.8, SE = 7.5) from which the members of an experimental colony (queen and four workers) originated did not explain variation in brood production among the experimental colonies and it was disregarded in the analyses below (Spearman's correlation: ‘on dose’ brood *vs.* source colony size, *ρ* = −0.10, *N* = 60, *P* = 0.44; ‘off dose’ brood *vs.* source colony size, *ρ* = 0.07, *N* = 60, *P* = 0.59; total brood *vs.* source colony size, *ρ* = −0.01, *N* = 60, *P* = 0.91).

We tested for dose-dependent brood production, timing of oviposition and food consumption during each period of the pulsed exposure using Spearman's correlation analyses. We tested for dose-dependent recuperation by analysing the differences in performance in experimental colonies between the ‘on dose’ and ‘off dose’ periods as follows. For a given variable *X*, denote the ‘on dose’ performance of a colony by *X_on_* and its ‘off dose’ performance by *X_off_*. For each colony we calculated (*X_off_* − *X_on_*), so that a positive value indicates that a colony produced more brood during the ‘off dose’ period, i.e. it showed recuperation. We investigated recuperation by testing whether (*X_off_* − *X_on_*) increased with imidacloprid dosage using Spearman's correlation analysis.

For brood production, once the statistical significance of the dose-response relationship was established by correlation we used Bayesian Hierarchical Models (BHM) to fit a relationship between ‘brood’ and ‘dosage’. In each BHM, we fitted: *brood* ∼ Poisson(*μ*); log(*μ*) ∼ *α* + *β* × log(*dosage*+1)+*λ*. Here, *α* and *β* are fitted coefficients analogous to the conventional regression coefficients of slope and intercept, and *λ* is a ‘random effects’ term to accommodate overdispersion (*λ* has a normal distribution with a mean of zero). Each model was fitted with 40,000 iterations of Bayesian inference using a Markov Chain-Monte Carlo method with Gibbs sampling after a burn-in period that discarded the first of 7000 iterations on each chain. We obtained confidence intervals on this relationship as follows. The pairs of *α* and *β* values from the final 40,000 iterations of the Bayesian inference estimate the posterior joint probability distribution of the two coefficients; we therefore plotted the 40,000 relationships corresponding to these pairs and extracted the upper and lower percentiles (2.5%, 97.5%) of the fitted brood values that corresponded to each imidacloprid intake across the range of interest. For brood production, we estimated the EC_50_ (half maximal effective concentration) and EC_10_ using the BHM best-fit relationships. We estimated EC values for the imidacloprid-induced reduction in food consumption by using GraphPad Prism v6.0c and evaluated the goodness of fit based on *R* ^2^. BHM procedures were implemented in WinBUGS v1.4.3 [39], while all other statistical analyses were conducted in R v3.0.0 [40].

## Results

In both pulsed and continuous exposure experiments, *B. terrestris* queens in experimental colonies began producing eggs after approximately two days and some brood progressed to a larval stage within the 14-day periods. No queens died during the experiments and there was negligible worker mortality (one dead worker at 98 ppb, two dead at 39 ppb in the same colony, two dead at 16 ppb in separate colonies).

**Figure 1:**
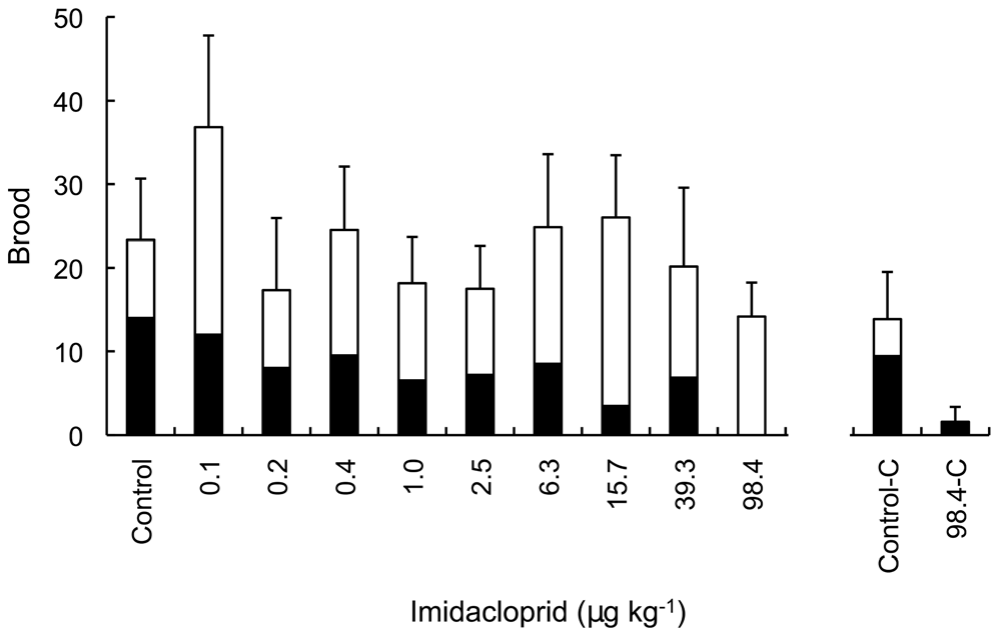
Brood production in *Bombus terrestris* colonies during a pulsed or continuous exposure to imidacloprid. Mean number of brood produced in standardised *Bombus terrestris* colonies (*N* = 60) during 28-day pulsed or continuous exposure to dietary imidacloprid. For pulsed exposure (from left to right, ‘Control’ to ’98.4’): brood produced during the 14-day ‘on dose’ period (black bars), during which colonies were exposed to imidacloprid in syrup at the specified dosage (in µg kg^−1^  =  parts per billion); and brood produced during the subsequent 14-day ‘off dose’ period (white bars), during which all colonies fed exclusively on control syrup. For continuous exposure (‘Control-C’ and ’98.4-C’): brood produced during first 14 days of exposure (black bars) and brood produced during second 14 days of exposure (white bars). Where a column does not contain a black bar or a white bar, zero brood were produced during days 1–14 or days 15–28, respectively. Error bars indicate ± SE of mean brood production over 28 days.

**Figure 2:**
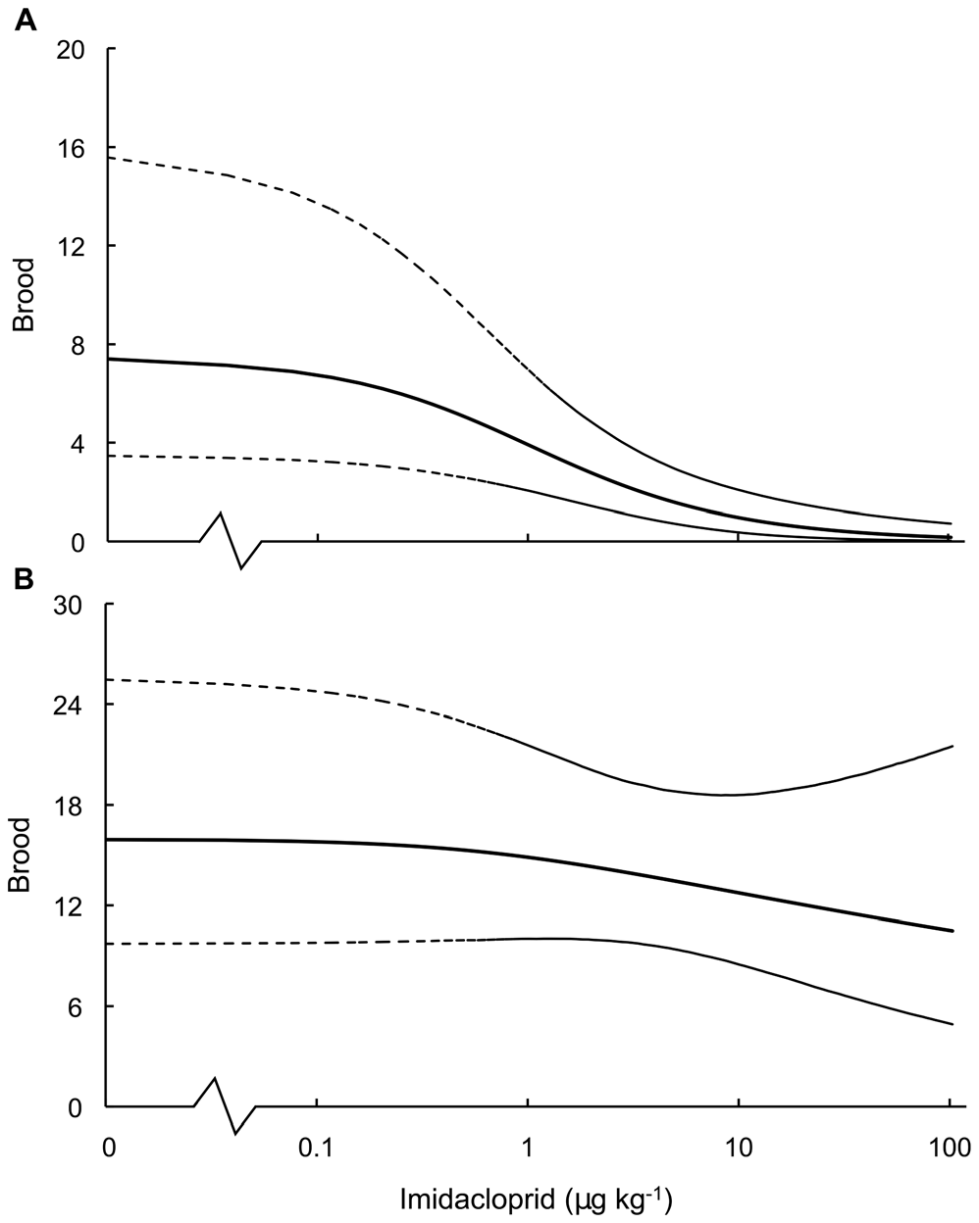
Best-fit dose-response relationships of brood production in *Bombus terrestris* colonies under pulsed exposure to imidacloprid. Dose-response relationships of brood production in standardised *Bombus terrestris* colonies (*N* = 60) following a 28-day pulsed exposure to dietary imidacloprid in syrup. Specifically, (A) brood production during the 14-day ‘on dose’ period of pulsed exposure in which bees fed on syrup dosed with imidacloprid and (B) total brood production taken over the entire 28-day pulsed exposure (including brood produced during the 14-day ‘on dose’ period and during the subsequent 14-day ‘off dose’ period in which imidacloprid was removed from the bees' diet). Solid lines indicate the best-fit dose response relationship (obtained using Bayesian Hierarchical Modelling of the data summarized in Figure 1, see Methods) and dashed lines indicate the relationship's 95% confidence intervals.

During the 14-day ‘on dose’ period of pulsed exposure, colonies exhibited dose-dependent repression of brood production such that fewer brood were produced as dosage increased up to 98 ppb imidacloprid (Spearman's correlation: ‘on dose’ brood *vs.* dosage, *ρ* = −0.45, *N* = 60, *P*<0.001; Figure 1). The dose-response relationship for brood and imidacloprid dosage during the ‘on dose’ period was given by *brood*  =  exp[2.002−1.788×log(*dosage*+1)] and the standard deviation of the overdispersion parameter was SD(*λ*) = 1.89 (Figure 2). Based on this relationship, the EC_50_ and EC_10_ values for imidacloprid's affect on brood production were 1.44 ppb and 0.15 ppb, respectively.

**Figure 3:**
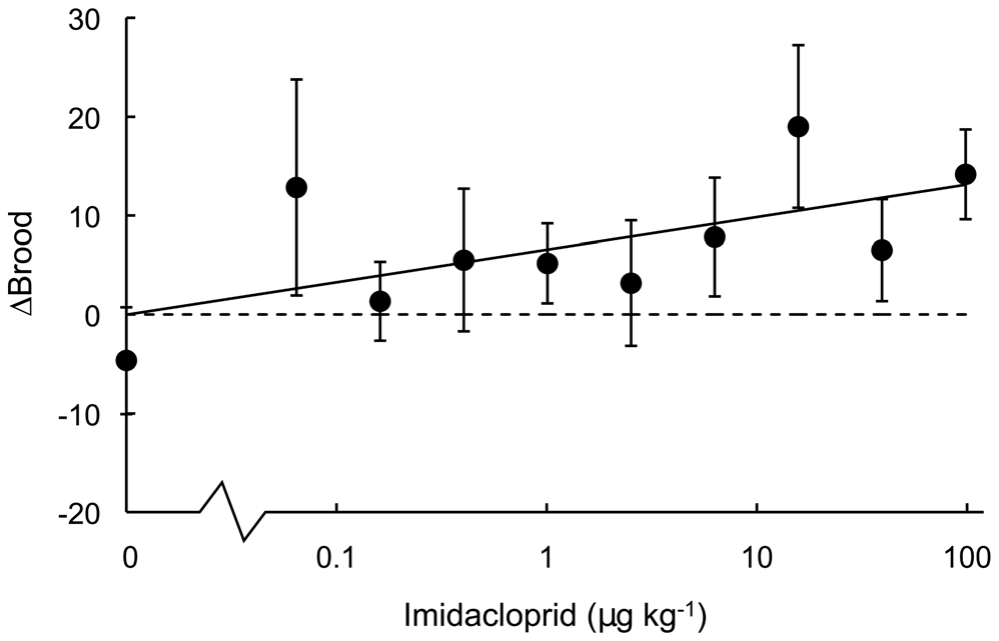
Recuperation of brood production in *Bombus terrestris* colonies during a pulsed exposure to imidacloprid. Recuperation of brood production in standardised *Bombus terrestris* colonies (*N* = 60) during the 14-day ‘off dose’ period of pulsed exposure, wherein bees fed exclusively on undosed control syrup. The ‘off dose’ period followed a 14-day ‘on dose’ period during which bees' fed on syrup dosed with imidacloprid at the given concentrations (in µg kg^−1^  =  parts per billion). Recuperation (*ΔBrood*) is determined by analyzing the difference in brood production between the ‘on dose’ (days 1–14) and ‘off dose’ (15–28) periods, specifically: *ΔBrood*  =  *Brood_off_* − *Brood_on_*, with a positive value indicating increased production of brood when ‘off dose’. Data represent the means and error bars indicate ± SE. The solid line indicates the following logarithmic trend: *ΔBrood* = 1.428 × ln(*dosage*) + 6.533, *R^2^* = 0.38. Dashed line indicates *ΔBrood* = 0.

During the 14-day ‘off dose’ period, brood production showed dose-dependent recuperation (Spearman's correlation: (*Brood_off_* − *Brood_on_*) *vs.* dosage, *ρ* = 0.32, *N* = 60, *P* = 0.01; Figure 3). Dosage did not significantly affect brood production during the ‘off dose’ period (Spearman's correlation: ‘off dose’ brood *vs.* dosage, *ρ* = 0.10, *N* = 60, *P* = 0.47; Figure 1) and, taken over the entire 28-day pulsed exposure, total brood production was not significantly correlated with imidacloprid dosage (Spearman's correlation: total brood *vs.* dosage, *ρ* = −0.13, *N* = 60, *P* = 0.32; Figure 1). However, we note that based on the 28-day dose-response relationship for brood and imidacloprid, given by *brood*  =  exp[2.770−0.198×log(*dosage*+1)] with SD(*λ*) = 1.25 (Figure 2), recuperation of brood production was incomplete at higher dosages. For example, a 32% reduction remained apparent in colonies dosed with imidacloprid at 98 ppb (Figure 2). The EC_50_ value for reduced brood production over the entire 28-day pulsed exposure was beyond our tested dosage range (>98 ppb), while the EC_10_ was estimated at 2.5 ppb.

Based on the fitted dose-response relationships (Figure 2), we estimate that 14-day exposures to dietary imidacloprid at environmentally realistic levels of between 0.3 ppb and 10 ppb may reduce brood production in *B. terrestris* colonies by between 18–84% (Table 1). However, the effects of recuperation in this residue range are such that given a further 14 days without exposure the drop in brood is ameliorated to between 2–19% (Table 1).

**Table 1:** Estimated decrease in brood production exhibited by *Bombus terrestris* colonies during pulsed exposure to realistic imidacloprid residues, equivalent to those previously detected in nectar of treated crops.

Realistic exposure scenario	Imidacloprid residue (ppb)	14-day ‘on dose’ brood reduction (%)^a^	28-day pulsed exposure brood reduction (%)^b^
OSR–Europe^c^	0.3	18 (14–24)	2 (0–6)
OSR–USA^c^	0.8	37 (30–45)	5 (0–12)
Mean max. level^d^	1.9	56 (51–64)	9 (0–19)
Gill et al.^e^	10.0	84 (84–86)	18 (9–27)

Reductions are relative to the number of brood produced in undosed control colonies and were obtained using the appropriate BHM best-fit dose-response relationship from Figure 2. The reduction's 95% confidence intervals, given in parentheses, were also obtained from BHMs in Figure 2.

a Refers to the estimated decrease in brood production expected after a 14-day exposure to imidacloprid at the given dosage.

b Refers to the estimated total decrease in brood after a 28-day pulsed exposure at the given dosage (14 days ‘on dose’, 14 days ‘off dose’).

c Maximum imidacloprid residues detected in the nectar of oilseed rape [21]. Data originates from studies conducted only in Member States of the European Union (OSR–Europe) and from studies including North America (OSR–USA).

d Mean maximum level of neonicotinoid residues in nectar calculated from 20 studies [56].

e Residues in dosed syrup used in a semi-field trial conducted by Gill et al. [8].

Recuperation is unlikely to be attributable to acclimation over time because brood production remained repressed under continuous exposure at 98.4 ppb over 28 days (Figure 1). Specifically, colonies dosed at 98.4 ppb imidacloprid exhibited significantly reduced brood production over 28-days compared to control colonies (ANOVA: dosage, *F*_1, 21_ = 6.33, *P*<0.05), but brood production did not differ between successive 14-day periods (days 1–14 and 15–28) of continuous exposure (ANOVA: period, *F*_1, 21_ = 2.22, *P* = 0.15).

Where brood were produced, imidacloprid did not affect the timing of first oviposition during the ‘on dose’ period (Spearman's correlation: days until oviposition *vs.* dosage, *ρ* = 0.11, *N* = 35, *P* = 0.5; Table 2), but it delayed oviposition in the subsequent ‘off dose’ period (Spearman's correlation: days until oviposition *vs.* dosage, *ρ* = 0.53, *N* = 45, *P*<0.001; Table 2).

**Table 2:** Mean number of days taken by *Bombus terrestris* queens to undertake oviposition during pulsed exposure to dietary imidacloprid.

Imidacloprid dosage (µg kg^−1^ = ppb)	On dose: day of first oviposition (± SE)	Off dose: day of first oviposition (± SE)
Control	4.2 (1.1)	1.3 (0.3)
0.1	2.6 (1.1)	2.8 (0.9)
0.2	5.0 (1.5)	6.0 (1.9)
0.4	2.8 (1.2)	1.5 (0.4)
1.0	3.0 (1.3)	4.2 (1.4)
2.5	10.3 (0.3)	6.0 (2.1)
6.3	3.8 (1.9)	6.0 (2.1)
15.7	11.0 (0.0)	5.7 (1.6)
39.4	2.3 (1.0)	7.8 (1.7)
98.4	–a	7.2 (1.2)

Oviposition occurred in standardised experimental colonies (queen and four workers) during either the 14-day ‘on dose’ period of pulsed exposure (during which bees fed on syrup dosed with dietary imidacloprid at the given concentration) or the subsequent 14-day ‘off dose’ period (when all imidacloprid dosages were removed from the bees' diet).

a Oviposition did not occur during the ‘on dose’ period in colonies exposed at 98.4 ppb.

**Figure 4:**
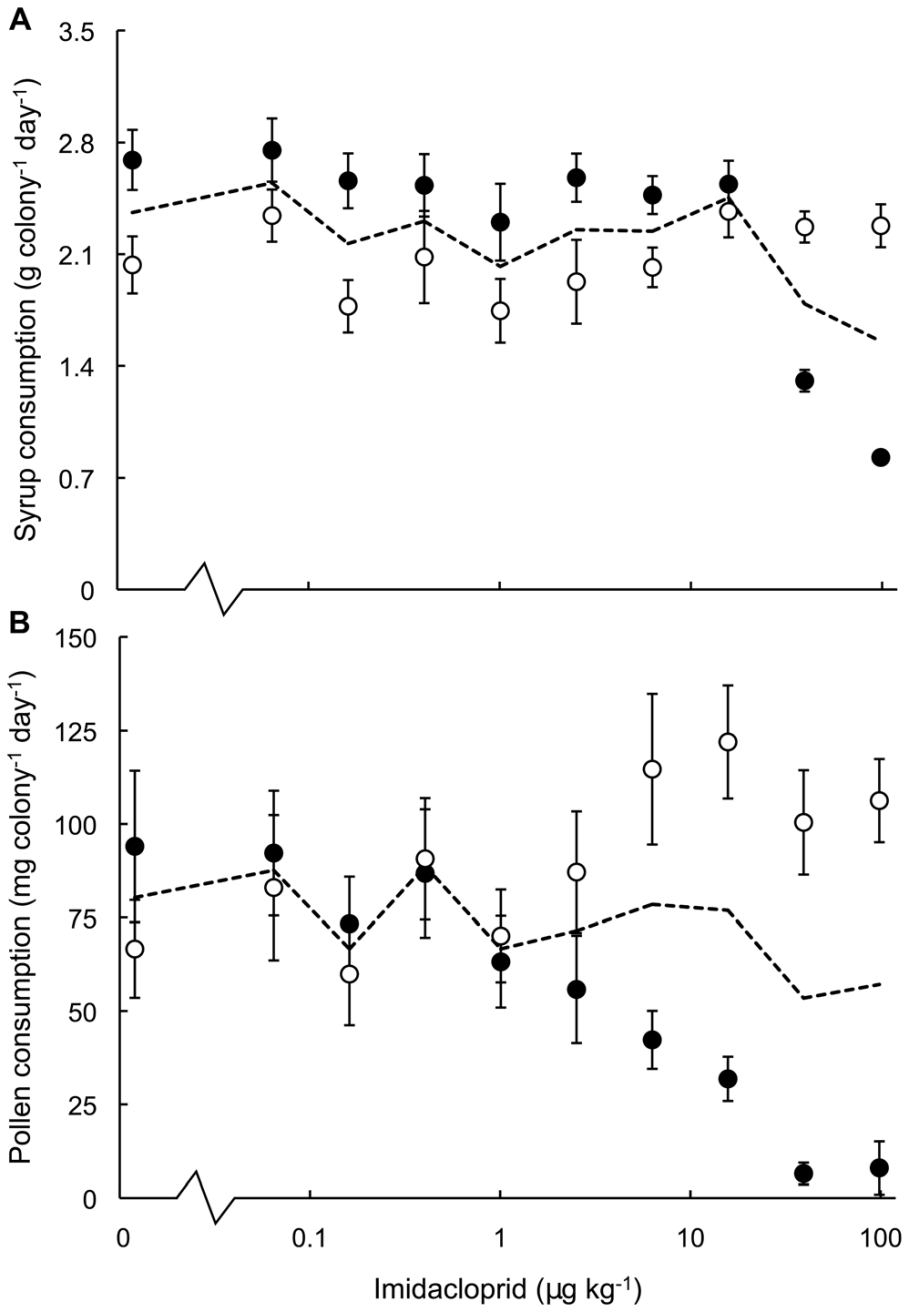
Food consumption in *Bombus terrestris* colonies during a pulsed exposure to imidacloprid. Feeding responses of standardised *Bombus terrestris* colonies (*N* = 60) during a 28-day pulsed exposure to dietary imidacloprid. Specifically, (A) mean daily syrup and (B) mean daily pollen consumption during the initial 14-day ‘on dose’ period feeding on imidacloprid dosed syrup (filled circles) and during the subsequent 14-day ‘off dose’ period feeding on undosed control syrup (unfilled circles). Dashed lines connect the mean consumption rates of colonies over the entire 28-day pulsed exposure. Error bars indicate ± SE. Control data (zero µg kg^−1^) are displayed slightly displaced on the x-axis for ease of inspection.

During pulsed exposure, we observed dose-dependent reductions in the daily consumption of syrup and pollen by experimental colonies whilst they were ‘on dose’ (Spearman's correlation: ‘on dose’ syrup consumption *vs.* dosage, *ρ* = −0.59, *N* = 60, *P*<0.001; ‘on dose’ pollen consumption *vs.* dosage, *ρ* = −0.77, *N* = 60, *P*<0.001; Figure 4). Based on these results, the EC_50_ and EC_10_ values for reduced pollen consumption were 4.4 ppb (*R* ^2^ = 0.95) and 0.2 ppb (*R* ^2^ = 0.96), respectively, while the equivalent values for reduced syrup consumption were >98 ppb (*R* ^2^ = 0.90) and 23.6 ppb (*R* ^2^ = 0.97).

During the ‘off dose’ period, colonies demonstrated dose-dependent recuperation of both syrup consumption (Spearman's correlation: (*Syrup_off_* − *Syrup_on_*) *vs.* dosage, *ρ* = 0.60, *N* = 60, *P*<0.001) and pollen consumption (Spearman's correlation: (*Pollen_off_* − *Pollen_on_*) *vs.* dosage, *ρ* = 0.81, *N* = 60, *P*<0.001). Dosage did not significantly affect syrup consumption during the ‘off dose’ period (Spearman's correlation: ‘off dose’ syrup consumption *vs.* dosage, *ρ* = 0.21, *N* = 60, *P* = 0.11; Figure 4), but pollen consumption significantly increased among colonies previously exposed to higher dosages (Spearman's correlation: ‘off dose’ pollen consumption *vs.* dosage, *ρ* = 0.40, *N* = 60, *P* = 0.001; Fig. 4).

Taken over the entire 28-day pulsed exposure period, the amount of syrup and pollen consumed in experimental colonies declined as imidacloprid dosage increased (Spearman's correlation: syrup consumption *vs.* dosage, *ρ* = −0.47, *N* = 60, *P*<0.001; pollen consumption *vs.* dosage, *ρ* = −0.25, *N* = 60, *P* = 0.05; Figure 4), demonstrating that recuperation of food consumption was incomplete. From these results, EC_50_ values were calculated to be 43.7 ppb (*R* ^2^ = 0.50) for reduced pollen consumption and >98 ppb (*R* ^2^ = 0.68) for reduced consumption of syrup, while EC_10_ values were 16.2 ppb (*R* ^2^ = 0.60) and 32.4 ppb (*R* ^2^ = 0.78) for pollen for syrup, respectively.

After using partial correlation analysis to control for the effects of dosage, brood production in experimental colonies increased with higher daily consumption of both syrup and pollen (Pearson's partial correlation: brood *vs.* syrup consumption, *r* = 0.32, *df* = 58, *P* = 0.01; brood *vs.* pollen consumption, *r* = 0.59, *df* = 58, *P*<0.001).

## Discussion

Under pulsed exposure to dietary imidacloprid, standardized colonies of *B. terrestris* bumble bees ‘on dose’ for 14 days exhibited dose-dependent repression of brood production, such that their productivity decreased as dosage increased up to 98 ppb. The removal of imidacloprid from colonies during the subsequent 14-day ‘off dose’ period produced dose-dependent recuperation of brood production to the extent that total productivity under pulsed exposure was not correlated with dosage up to 98 ppb. Pulsed exposure of colonies to dietary imidacloprid at 98 ppb produced the largest observed recuperation, but continuous exposure to the same concentration repressed brood production without recuperation during a separate experiment of equal duration. We therefore argue that recuperation is primarily achieved by the reversibility of imidacloprid-induced effects rather than acclimation to imidacloprid over time.

The dose-dependent decrease in brood production we observed in queenright colonies mirrors the effect on brood production in queenless microcolonies of *B. terrestris* workers over the same period of time [28]. Similarly, our EC_50_ value for a 14-day exposure (1.44 ppb) is comparable to the EC_50_ for imidacloprid's effect on drone production in *B. terrestris* microcolonies exposed over eleven weeks (3.7 ppb) [26]. However, the recuperation of brood production in bumble bee colonies we observed under pulsed exposure is a new finding. Other insects show recuperation from some imidacloprid-induced effects during pulsed exposure [35]–[38], but we are the first to demonstrate the resilience of an important demographic endpoint in bees. In our study, when imidacloprid exposure ceased, the ameliorating effect of recuperation on bumble bee brood production was such that the EC_50_ for a 28-day pulsed exposure was raised beyond 98 ppb. However, we note that recuperation remained incomplete at higher doses, with overall brood productivity still reduced by between 19–32% at dosages between 10–98 ppb. According to a recent guidance document for the risk assessment of plant protection products on bees [41], a reduction in this range would constitute a ‘medium’ colony-level-impact and could translate into a similar effect on colony size. Additionally, we found that oviposition was delayed during the ‘off dose’ period of pulsed exposure in colonies that were first presented with imidacloprid at higher dosages. Our results suggest that where bumble bees experience a pulsed exposure to residues of imidacloprid above 10 ppb [25], incomplete recuperation of brood production and delayed oviposition could detrimentally impact colony size and thereby influence colony fitness [30], [31].

Consumption of syrup and pollen in our experimental colonies also underwent dose-dependent repression and recuperation during the ‘on dose’ and ‘off dose’ periods of pulsed exposure, respectively. Repression was most severe in pollen consumption, with an EC_50_ of just 4.4 ppb, and both feeding endpoints showed incomplete recuperation at the two highest dosages (39 and 98 ppb). This result is somewhat consistent with a previous study of recovery in honey bees, in which recuperation of foraging activity was incomplete in colonies exposed to imidacloprid at 48 ppb [34]. Since the pollen in our experiment was not dosed, the imidacloprid in the syrup reduced the bees' overall ability or desire to feed during the ‘on dose’ period. In a previous study, *B. terrestris* workers exposed to dietary imidacloprid in microcolonies exhibited dose-dependent feeding reductions that were also linked to reductions in brood productivity [28]. Consequently, it was hypothesized that imidacloprid-induced nutrient limitation might play some part in repressing bumble bee egg production during exposure [28]. Our data lends support to this hypothesis because it demonstrates that: a) queenright colonies that consumed more syrup and pollen produced more brood; b) bees showed dose-dependent reductions in feeding whilst ‘on dose’; c) repression of brood production coincided with repressed feeding. Additionally, recuperation of food consumption and brood production in colonies occurred simultaneously when exposure ceased and we therefore suggest that removal of imidacloprid from the bees' diet caused feeding rates to recover, which re-established sufficient nutrient intake to facilitate reproduction in bumble bee queens. Although the mechanism for recuperation of food consumption was not studied here, we speculate that it has its basis in the metabolic elimination of the toxicant [42], which in a previous study appeared to take place within 48 hours in bumble bees fed imidacloprid at 98 ppb [43].

### Comparison with results of semi-field trials

In our study, a two-week exposure to dietary imidacloprid at 10 ppb in syrup substantively reduced brood production in *B. terrestris* colonies. In a semi-field trial, Gill et al. [8] found that *B. terrestris* colonies also dosed with 10 ppb imidacloprid solely in artificial nectar produced significantly fewer workers at the end of a four-week exposure, without suffering elevated levels of in-colony worker mortality. Although they did not measure egg production, Gill et al. found that imidacloprid-dosed colonies accumulated fewer larvae and pupae over 4 weeks and speculated that this was due to imidacloprid's effect on brood survival. Based on our findings, we hypothesize that repressed brood production may have been an important cause of Gill et al.'s observations.

In a second semi-field study, Whitehorn et al. [9] exposed *B. terrestris* colonies to field-realistic dosages of dietary imidacloprid for two weeks in the laboratory and monitored colony development for a further six weeks in the field. We exercise caution when comparing our observations to Whitehorn et al.'s because pollen was their principle delivery vehicle for imidacloprid. However, following a similar exposure duration and an extended imidacloprid-free period, Whitehorn et al. found no significant effect of imidacloprid on the number of pupae and workers in colonies, but a strong negative effect on the number of queens. Potentially, recuperation of brood and worker production occurred in Whitehorn et al.'s colonies when exposure ceased, but for some unknown reason any recovery was insufficient to sustain normal levels of queen production. Their observations may originate in either increased intoxication of the existing queen caused by consumption of contaminated pollen during lab exposure or the impact of a longer exposure to imidacloprid in the stored nectar and pollen within in the nest, which is important for successful development of new queens [44]. Additionally, if imidacloprid reduces the foraging efficiency of workers [8] then exposed colonies may lack sufficient resources to produce the normal quota of queens, each of which comprises almost twice the biomass of a male bumble bee [30]. Furthermore, brood and worker production in bumble bee colonies may recover better following imidacloprid exposure than other important endpoints. We therefore suggest that the potential for recuperation of performance in demographically important endpoints other than brood production is an area requiring further research in bumble bees.

### Environmental relevance

Whilst our study raises further concerns about the threat to wild bumble bees from imidacloprid it also indicates some resilience to a pulsed exposure that could arise during the synchronized bloom of a treated mass-flowering crop. However, when interpreting the environmental relevance of our findings we recognize the limitations of our study, which are as follows. First, the pollen consumed in our colonies was not dosed. There is no reason to suspect different levels of toxicity arising due to ingestion of imidacloprid in nectar *vs*. pollen, but a bumble bee queen is likely to eat a substantial pollen load whilst producing eggs [45] and consequently her exposure in the wild may be more severe than tested here.

Second, the duration of exposure in the environment may differ from our experiment. Exposure for 14 days is a reasonable first approximation because, for example, roughly 75% of the flowering of winter-sown oilseed rape in the UK occurs over a peak period of about two weeks [32]. However, total flowering duration can extend across five weeks or more and bumble bee colonies may continue to forage on mass-flowering crops throughout their blooming period [46]. Conversely, colonies will vary in the extent to which their development intersects with the blooming period of mass-flowering crops because bumble bee queens emerge from their overwinter sites and initiate colonies at various times in spring [47]. Consequently, colonies of later-emerging queens may develop after the crop's bloom has largely or completely declined and could broadly escape neonicotinoid effects.

Third, our study may underestimate the severity of imidacloprid's effects. For example, we focus primarily on brood production, but there are other demographically important endpoints such as mortality. A diet dosed with imidacloprid at realistically high levels (10 ppb) appears to raise mortality in colonies by increasing the risk that workers become lost whilst foraging and in addition exposed foragers tend to return to the nest with less pollen less often [8]. If these impacts also occur at lower dosages (<10 ppb), which are more typically found in environmental nectar and pollen [21], they could certainly add to the stress on wild bumble bee colonies and diminish their reproductive output. Additionally, while the amount of brood and workers produced in a bumble bee colony can influence the quantity of new queens and males that are produced [30], [31], the quality of sexual offspring produced may also be critical for colony fitness. For example, body mass predicts whether a young queen will survive diapause [48] and body size may impact on a male's mating success [49]. Furthermore, wild colonies are likely to be under additional stresses from pathogens [50], parasites [51] and other agrochemicals [8], which could augment the severity of a neonicotinoid's impact and the potential for recovery. Additive [8] and synergistic [52] effects of certain neonicotinoids and other agrochemicals have been reported for bees, but further study into combinatorial effects of neonicotinoids and other potential stressors is necessary. Finally, under laboratory conditions winter honey bees appear to be less sensitive to imidacloprid than summer honey bees [53]. Although winter active bumble bees have been observed at latitudes as far north as southern England [54], unlike winter honey bees they are unlikely to be social foragers because bumble bee colonies typically perish in the autumn before newly mated queens enter hibernation [55]. Therefore, if seasonal differences in sensitivity exist in wild bumble bees, foragers from spring and late summer colonies would have to be compared. Commercially bred bumble bees, which were used in autumn and winter in our current study, are produced throughout the year. As these bees are reared under standardised conditions, it is unlikely that they would show seasonal variation in sensitivity to imidacloprid. However, the effects reported here could be more severe in wild colonies and in future work it would be important to compare the sensitivity of commercially reared and wild bumble bees.

## Conclusions

Our study provides further evidence that dietary neonicotinoid pesticides in the environmentally realistic range can have detrimental effects on bumble bee health, specifically by repressing brood production and nutritional intake in queenright colonies. We also show, however, that bumble bees may be somewhat resilient to a pulsed exposure because they exhibit dose-dependent recuperation of brood production when exposure ends. We acknowledge that to interpret the environmental relevance of our findings for wild bumble bee colonies additional studies are necessary. These should seek to establish whether recuperation from pulsed exposure to neonicotinoids occurs during extended exposures and for other demographically important endpoints besides brood production. Finally, the severity of imidacloprid's impact on bumble bees appears to be highly sensitive to its dietary level even within the currently recognized environmentally realistic range [21]. Unfortunately, this range is based on scant published data [56] and more widespread surveys of residues in crops and colonies, such as those recently begun in the USA [25], are therefore urgently required.
